# “Don’t Mind If I Do”: The Role of Behavioral Resistance in Self-Control’s Effects on Behavior

**DOI:** 10.3389/fpsyg.2020.00396

**Published:** 2020-03-13

**Authors:** Marleen Gillebaart, Floor M. Kroese

**Affiliations:** Department of Social, Health, and Organizational Psychology, Utrecht University, Utrecht, Netherlands

**Keywords:** self-control, behavioral resistance, task aversiveness, goal-directed behavior, health, sustainability

## Abstract

High self-control is known to be related to the performance of behaviors that have long-term benefits, such as healthy eating. Recently, studies have suggested that people with high self-control may perform goal-directed behaviors not by exerting effortful control but rather by employing smart, effortless strategies. The current paper investigates the crucial role of behavioral resistance in the relation between self-control and goal-directed behaviors: we propose that people with high self-control feel less resistance toward goal-directed behaviors compared to people with low self-control, and that this is associated with the increased frequency of performing these behaviors. Three cross-sectional studies were conducted in which participants reported on their level of self-control, behavioral resistance toward behaviors in the sustainability, healthy eating, exercise, and study/work domains, and their behavior in those domains. Findings consistently show that the relation between self-control and various behaviors is indeed partially mediated by behavioral resistance, although the study designs preclude establishing causal relations. It is implied that lower resistance makes it easier for people with higher self-control to perform the goal-directed behaviors, without requiring much effort. This notion yields an interesting, novel perspective on how people with high self-control manage to function so well.

## Introduction

Unfortunately, only doing things you feel like doing does not tend to result in achieving long-term goals. In fact, people’s daily lives are filled with instances in which they need to employ self-control to overcome initial resistance or inhibit hedonic impulses in order to align their behavior with their long-term goals (e.g., choosing between an apple or a candy bar as an afternoon snack, resisting the urge to stay on the couch and watch TV in order to go out and exercise at night, doing homework instead of playing video games). However, everyone has that self-disciplined colleague, friend, acquaintance, or family member that seems to simply *not mind* performing the “ought to” behaviors that come with pursuing our long-term goals: they go to the gym a few times a week without complaining, they skip the office cake during coffee breaks without seeming to care so much, and they seem to always arrive on time without any effort. These people are most likely people with a high level of trait self-control, a characteristic that is known as the ability to resist temptations and pursue long-term goals. However, they do not fit the image of someone effortfully dealing with conflicts or dilemmas. Instead, they seem to effortlessly perform self-regulatory behaviors that in the end lead them to their long-term goals, and, most importantly, they do not seem to mind. In this paper, we introduce *behavioral resistance* as a mediating factor in the relation between trait self-control and performance of long-term goal-directed behaviors. Behavioral resistance can be defined as the extent to which people perceive the behaviors or actions they need to perform in order to reach their goal as unpleasant, and feel a literal sense of resistance with regards to that behavior. That is, we propose that people with high trait self-control feel less behavioral resistance toward these behaviors, which in turn (partially) explains their good self-regulatory performance.

Self-control is considered an essential human characteristic that allows us to forego immediate gratifications for delayed ones, and is a necessary ability for achieving long-term goals of health, wellbeing, interpersonal relationships, and performance (Metcalfe and Mischel, 1999; Tangney et al., 2004; De Ridder et al., 2012). Until recently, self-control was defined solely as the effortful inhibition of impulsive behaviors and predominant responses (Baumeister et al., 1998; Muraven and Baumeister, 2000). Research on self-control has therefore focused mainly on how this specific type of self-control operates. However, this narrow perspective on self-control does not take into account the notion that many people are actually successful in resolving subsequent self-control dilemmas in daily life: higher levels of trait self-control predict successful work and academic outcomes (Tangney et al., 2004; Duckworth and Seligman, 2005), better health (Moffitt et al., 2011), greater happiness (Cheung et al., 2014; Hofmann et al., 2014), and better interpersonal relationships (Vohs et al., 2011).

People with high self-control are generally relatively successful in resolving self-control dilemmas in such a way that they yield self-regulatory benefits and remain *en route* to their long-term goal. However, if one were to expend effort with each instance of self-control exertion, one would never reach that long-term goal due to fatigue or reduced motivation. It was recently suggested that people with high trait self-control may use certain strategies and automatic routines that do not actually rely on effortful inhibition, but instead make use of more effortless processes, such as more beneficial habits in the areas of diet, exercise, and studying (Adriaanse et al., 2014; Galla and Duckworth, 2015; Gillebaart and De Ridder, 2015; Gillebaart and Adriaanse, 2017). Indeed, people with high self-control report to experience fewer self-control conflicts in their daily lives (Hofmann et al., 2012). This new line of research into “smart” self-control brings about important questions to deepen our understanding of how successful self-control operates. Beyond demonstrating that people with high self-control perform well without exerting much effort, it is imperative to understand *why* they do not need to exert effort. As implicated by findings showing that people with high self-control experience less conflict as compared to people with low self-control when faced with typical self-control dilemmas (Hofmann et al., 2012; Gillebaart and De Ridder, 2015; Gillebaart et al., 2016), we propose that differences in experienced behavioral resistance may be what makes people with high self-control better at performing long-term congruent behaviors.

Behavioral resistance goes beyond simple self-control capacity, or availability of self-regulatory resources, by introducing how people experience the actions they need to undertake to get closer to their goal. Importantly, behavioral resistance, or the extent to which people perceive the behaviors they need to perform in order to reach their goal as unpleasant, is not related to their appreciation of the focal long-term goal, but rather of the means through which they are going to reach that goal: someone can be very positive about wanting to become physically fit, while at the same time dreading going to the gym. Similarly, one person may feel much more resistance toward the idea of having to study in the library compared to someone else, even though both strive for academic success. This difference is also what makes behavioral resistance distinct from concepts like intrinsic vs. extrinsic motivation, which relates to being motivated for goals rather than tasks, activities, actions, or behaviors in service of a pursued goal.

The concept of behavioral resistance is related to task aversiveness, a familiar phenomenon in research on procrastination (Watson, 2001; Steel, 2007; Kroese and De Ridder, 2016). Task aversiveness can be defined as the extent to which a task or behavior is perceived to be unpleasant or unenjoyable to perform (Milgram et al., 1988; Blunt and Pychyl, 2000), and is an important cause of procrastinating performance of different types of behaviors such as studying (Milgram et al., 1995) and working on personal projects (Blunt and Pychyl, 2000). Typically, the role of task aversiveness in the relation between self-control and the performance of behaviors has been explained such that more self-control is necessary when performing aversive tasks (Ainslie, 2013; Kurzban et al., 2013). Furthermore, the negative affect that may accompany feelings of resistance (Blunt and Pychyl, 2000) may further undermine long-term goal pursuit in people with low self-control, as they also show more trouble adequately regulating their emotions (Tice et al., 2001; Tangney et al., 2004). The relation between behavioral resistance and behavioral performance has been tested most explicitly in domains of academic and work behavior, but similar relations have been implicated to exist for health behavior (Kroese and De Ridder, 2016; Nauts et al., 2016). This perspective seems to be in line with notions of effortful self-control: it costs more effort to perform tasks that one finds aversive (Ainslie, 2013), and people with high self-control will be better able to exert this effort in an efficient way by choosing certain strategies (Hennecke et al., 2019).

The current paper proposes an additional role of behavioral resistance in the relation between self-control and long-term goal-directed behavior: we suggest that self-control success is partially explained by experiencing less resistance toward the behavior at hand. People with low and high self-control alike may have similar long-term goals and intentions (e.g., maintaining a healthy weight), but while those with low self-control constantly struggle to get over their resistance to perform the behaviors that are required to reach their goal (e.g., exercising), people who report a high level of self-control do not experience it as such: they feel less resistance, or aversion, toward the behaviors and are able to perform them without requiring effortful control.

The consideration of behavioral resistance in explaining self-control success is valuable especially in a framework of effortless self-control: if we move away from a classic conceptualization of self-control as inhibitory, effortful control, we also need to explore what sets high and low self-control apart. Elucidating this novel factor of interest in explaining successful self-control advances recent theorizing in this domain by providing further understanding into *why* people with high self-control can be successful in such effortless ways. Moreover, it may provide a new practical angle for self-control improvement and training, which is valuable considering the mixed results on more traditional ways of self-control training (Beames et al., 2017; Friese et al., 2017). The aim of the current research is therefore to investigate behavioral resistance as an underlying mechanism of self-control success by exploring the associations between (trait) self-control, resistance toward specific behaviors, and performance of behaviors in health-related areas.

In three studies, the indirect effect of self-control on several self-reported behaviors through behavioral resistance was investigated. In Study 1, we first set out to provide empirical proof of principle for the proposed mediation by studying sustainable behavior. In Study 2, associations were assessed between trait self-control, behavioral resistance, and frequency of three types of behaviors that are often associated with self-control: healthy eating behavior, exercise behavior, and study/work behavior. In Study 3, we conceptually replicated and improved Study 2 within the healthy eating and exercise domain. In all three studies, it was hypothesized that resistance would mediate between self-control and behavior: A higher level of self-control would predict a lower level of behavioral resistance, which would in turn increase frequency or amount of the desired, goal-directed, “ought to” behaviors. Due to the cross-sectional nature of the study designs, it must be noted that the causality directions of these associations cannot be established. These hypotheses, nor the studies, were preregistered. Power analyses were done based on previous studies establishing associations between self-control and (self-reported) behavior.

## Study 1

In this first study we aimed to provide a proof of principle on the notion that behavioral resistance toward self-control behaviors can (partially) explain the association between self-control and self-control behaviors. The study focused on the “sustainable behavior” domain, as a typical behavioral domain in which the required immediate behaviors (e.g., recycling, cutting back on meat consumption) are not immediately gratifying but serve a long-term goal. Many people have intentions to behave in a sustainable manner, but have trouble bridging the gap between intention and behavior (Kollmuss and Agyeman, 2002; Kennedy et al., 2009). Sustainable behavior often costs effort, and the short-term non-sustainable alternatives are often easier and thus more tempting. As such, sustainable behaviors have been linked to self-control in the literature (e.g., Ruepert et al., 2016; Redondo and Puelles, 2017).

Data from all studies was collected in collaboration with students. For educational purposes, additional variables were assessed. These variables were not of interest to the focus of the current paper. A list of additional variables assessed per Study is available upon request.

### Methods

#### Participants and Design

Participants were recruited for an online survey through social media. In total, 373 participants took part in the study, of which 76 participants were excluded who did not complete the whole survey or did not consent to processing of their data. Another 47 participants were excluded from analysis based on the fact that they lived with their parent(s)/guardian, a criterion that was deemed important since most of the sustainable behaviors tested referred to house-hold activities. The final sample consisted of 250 participants (86 males, 161 females, 3 other), with a mean age of 32.99 (*SD* = 18.19). The number of participants in the final sample was in accordance with the required sample (*N* = 115) size to find a small/medium effect in a mediation study with a power of 0.8 (alpha path 0.26, beta path 0.39; Fritz and MacKinnon, 2007).

The study had a cross-sectional design. The independent variable was self-control, the dependent variable was sustainable behavior, and the mediator was behavioral resistance. The study was approved by the institution’s ethics committee. Participants were not compensated for the study.

#### Materials

Gender, age, and living situation were assessed. Self-control was measured using the Brief Self-Control Scale (13 items; Tangney et al., 2004). An example item is: “I am good at resisting temptation.” The items were answered on a five-point Likert scale (1 = not at all, 5 = very much). Nine items were reversed so that a higher score on the scale indicated a higher amount of self-control. The reliability of the scale was good (Cronbach’s α = 0.80).

Sustainable behavior was assessed by the pro-environmental behavior scale (PBS; Whitmarsh and O’Neill, 2010), which contains 17 items. Car- and social-related items were removed, for the reasons that not everyone owns a car and the study does not examine social factors, respectively. There were 11 items left, which were all translated into Dutch. An example item is: “I avoid eating meat.” A four-point Likert scale was used to answers the items (0 = never, 3 = always). The reliability of the scale was good (Cronbach’s α = 0.72). The behavioral resistance scale was based on the same five activities that were measured in the PBS, namely: energy-saving behavior, travel choice, consumer behavior, recycling, and water-saving behavior. For each activity, participants were asked to rate “how unpleasant” they find doing it, on a scale from 1 (not at all unpleasant) to 7 (very unpleasant). The reliability of the scale was reasonable (Cronbach’s α = 0.66).

#### Procedure

Participants were recruited for the online survey via social media (e.g., Facebook, LinkedIn, WhatsApp). Participants were first asked to sign an informed consent. Next, demographic questions were assessed regarding gender, age, and living situation. After that, the self-control scale was presented, which was followed by the sustainable behavior scale. The sustainable behavior scale was followed by the behavioral resistance scale. The survey ended with a debriefing in which the goals and expectations of the study were laid out.

### Results

Means and standard deviations are presented in Table 1. Correlations demonstrated that self-control was significantly positively correlated to self-reported sustainable behavior [*r*_(250)_ = 0.22, *p* < 0.001], and significantly negatively correlated to behavioral resistance [*r*_(250)_ = −0.21, *p* = 0.001]. Behavioral resistance toward sustainable behavior and the self-reported performance of sustainable behaviors were significantly negatively correlated [*r*_(250)_ = −0.57, *p* < 0.001].

**TABLE 1:** Means and standard deviations for key variables in Study 1, Study 2, and Study 3.

	*M*	*SD*
**Study 1**		
Self-control	3.26	0.60
Behavioral resistance	2.88	0.75
Sustainable behavior	2.52	0.45
**Study 2**		
Self-control	3.08	0.60
Behavioral resistance healthy eating	3.23	1.07
Behavioral resistance exercise	3.66	1.35
Behavioral resistance study/work	3.62	1.41
Amount of healthy meals	4.87	1.40
Hours of exercise	2–4	
Hours of study/work	20–30	
**Study 3**		
Self-control	3.62	0.73
Behavioral resistance healthy eating	2.48	1.03
Behavioral resistance exercise	3.20	1.26
Fruit and vegetable intake days/week	3.10	2.30
Deep fried food intake days/week	1.75	1.67
Unhealthy snack intake days/week	3.85	2.34
Frequency of strenuous exercise days/week	<1	
Frequency of mild exercise days/week	∼1	
Minutes of physical activity/day	30–60	

**FIGURE 1:**
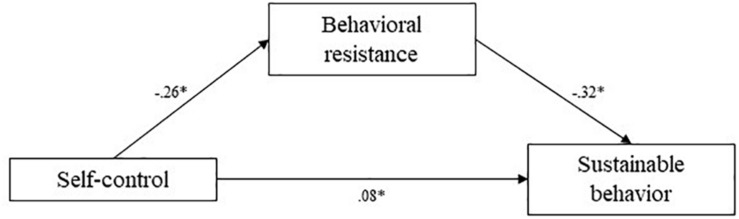
Study 1 mediation pathways including PROCESS macro (Hayes, 2012) unstandardized coefficients for associations between self-control, behavioral resistance, and sustainable behavior. ^∗^means: significant effect.

To test the hypotheses, mediation analyses were conducted using the Hayes (2012) PROCESS bootstrapping macro and guidelines for estimating indirect effects, with 1,000 bootstrap samples per analysis. Mediation pathways are depicted in Figure 1. The indirect effect of self-control on self-reported sustainable behavior through behavioral resistance was estimated at 0.08 (CI 95% [0.04; 0.14]). The confidence interval did not contain zero, therefore, this mediation pathway was significant. Self-control predicted lower levels of behavioral resistance, which in turn predicted higher levels of self-reported sustainable behavior, in line with our hypothesis.

### Discussion

This first study provides support for the notion that behavioral resistance plays an explanatory role between self-control and self-reports of sustainable behavior. A higher level of self-control predicted a lower level of behavioral resistance toward the sustainable behavior, which in turn increased the amount of desired sustainable behavior as reported by the participants. This suggests that people with high trait self-control experience less resistance, or aversion, to the behaviors they need to perform in order to achieve their long-term goals, which implies that they do not need to rely on effortful inhibition and initiation *per se* to overcome temptation and resistance, but rather do not mind these “ought to” behaviors as much as people with a lower level of self-control. On the other hand, people with lower levels of self-control have to overcome feelings of aversiveness when they want to carry out these goal-directed behaviors, which costs effort and would make tempting alternatives that offer immediate gratifications more appealing.

Continuing in this line of reasoning, we conducted Study 2 to expand from sustainable behavior to other self-control domains (i.e., healthy eating, exercising, and study/work behavior), as well as to further pinpoint the concept of behavioral resistance by developing a measure that goes beyond ratings of unpleasantness.

## Study 2

In this second study, the associations between self-control, behavioral resistance, and self-control behaviors in the health and academic/work domain were assessed via self-reports. The study focused on three types of behavior that are known to be prone to self-control dilemmas: healthy eating, exercising, and study/work behavior. The study had a cross-sectional design. The independent variable was self-control, the dependent variables were the self-control behaviors, and the mediator was behavioral resistance toward specific behaviors.

### Methods

#### Participants and Design

Participants were recruited for an online survey through social media. A total of 175 participants took part in this study. Due to missing data in the self-control and/or behavioral resistance questionnaires and the behavior questions on healthy eating, exercise, and study/work, 63 participants were excluded from the analyses, leaving a final sample of 112 participants. The number of participants in the final sample was nearly in accordance with the required sample (*N* = 115) size to find a small/medium effect in a mediation study with a power of 0.8 (alpha path 0.26, beta path 0.39; Fritz and MacKinnon, 2007).

The participant sample had a mean age of 30.85 (*SD* = 13.91) and consisted of 71 females, 40 males, and 1 participant who did not disclose their gender. The study was approved by the institution’s ethics committee. Participants did not receive compensation.

#### Materials

Self-control was assessed with the Brief Self-Control Scale (Tangney et al., 2004). Details of this scale can be found in the Materials section of Study 1. The scale proved reliable with a Cronbach’s α of 0.80.

Behavioral resistance was assessed separately for healthy eating behavior, exercise behavior, and study/work behavior. An adaptable behavioral resistance scale was constructed for this purpose. For each area of behavior, 10 items assessed how aversive participants felt toward the tasks that come with this behavior (e.g., for aversiveness toward healthy eating behavior: “Sometimes I don’t really feel like eating healthily,” for aversiveness toward exercise behavior: “I tend to delay getting ready to exercise,” for aversiveness toward study/work behavior: “Even if I don’t feel like it, I do my study work,” the latter being reverse coded). Per scale, 3 items were reverse coded. Answers were recorded on 7-point Likert scales ranging from 1 (not at all applicable to me) to 7 (very much applicable to me). All items can be found in Appendix. The scales proved reliable with a Cronbach’s α of 0.83 for behavioral resistance toward eating healthy, 0.90 for exercise behavior, and 0.93 for behavioral resistance toward study and work behavior.

To assess behavior, participants were asked to report how many times they ate a healthy meal per week (ranging from 0 to 7 times), how many hours a week they exercised (0–1, 1–2, 2–4, 4–6, or more than 6), and how many hours a week they spent on their studies or work (0–10, 10–20, 20–30, 30–40, or more than 40 h per week).

#### Procedure

Participants were invited to join an online survey through social media. Informed consent was obtained at the start of the survey. After filling out the Brief Self-Control Scale, for each behavior, the behavioral resistance scale, and questions about the actual behavior (healthy eating, exercise, and study and work behavior) were presented to the participants. Finally, demographic information was obtained, and participants were debriefed.

### Results

**FIGURE 2:**
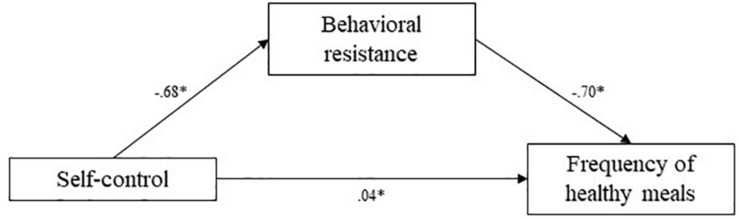
Study 2 mediation pathways including PROCESS macro (Hayes, 2012) unstandardized coefficients for associations between self-control, behavioral resistance, and frequency of healthy meals. ^∗^means: significant effect.

**FIGURE 3:**
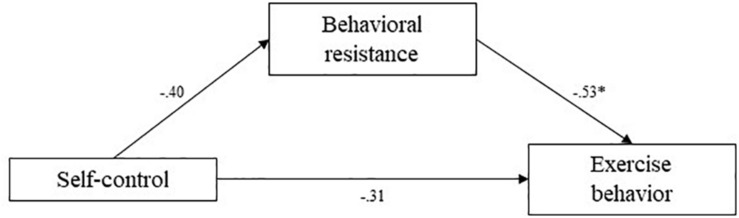
Study 2 mediation pathways including PROCESS macro (Hayes, 2012) unstandardized coefficients for associations between self-control, behavioral resistance, and exercise behavior. ^∗^means: significant effect.

**FIGURE 4:**
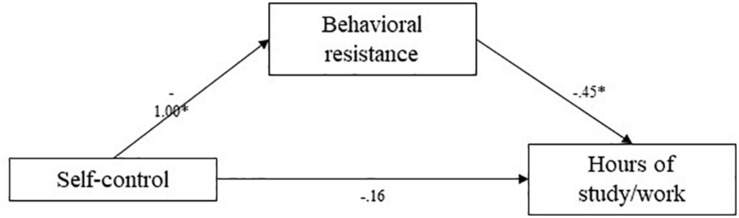
Study 2 mediation pathways including PROCESS macro (Hayes, 2012) unstandardized coefficients for associations between self-control, behavioral resistance, and hours of study/work. ^∗^means: significant effect.

Means and standard deviations for key variables are presented in Table 1. To test the hypotheses, mediation analyses were conducted using the Hayes (2012) PROCESS bootstrapping macro and guidelines for estimating indirect effects, with 1,000 bootstrap samples per analysis. Mediation pathways are presented in Figures 2–4.

#### Healthy Eating

In line with hypotheses, correlations between the key variables showed that self-control level was significantly positively related to self-reported frequency of healthy meals [*r*_(110)_ = 0.22, *p* = 0.02], and significantly negatively related to behavioral resistance toward eating healthy [*r*_(110)_ = −0.39, *p* < 0.001]. Behavioral resistance and reported frequency of healthy meals were negatively correlated [*r*_(110)_ = −0.54, *p* < 0.001]. These associations suggested that, as hypothesized, behavioral resistance may mediate between self-control and healthy eating behavior as reported by participants.

In line with hypotheses, analyses demonstrated a significant indirect effect of self-control and frequency of healthy meals through behavioral resistance toward eating healthy. The indirect effect was estimated at 0.48, and the 95% confidence interval of this indirect effect did not include zero (CI 95% [0.20;0.84]). This indicates that higher self-control predicts more healthy meals through lowered behavioral resistance.

#### Exercise Behavior

Unexpectedly, correlations between exercise behavior variables showed that self-control level was not significantly related to the self-reported amount of exercise [*r*_(110)_ = −0.05, *p* = 0.64], but marginally significantly negatively related to behavioral resistance toward exercising [*r*_(110)_ = −0.18, *p* = 0.056]. Behavioral resistance toward exercising and self-reported amount of exercise were significantly negatively related [*r*_(110)_ = −0.54, *p* < 0.001]. Despite the non-significant correlations, we formally tested mediation of behavioral resistance to test the hypotheses.

The indirect effect of self-control on amount of exercise through behavioral resistance toward self-reported exercise behavior was estimated at 0.21. The 95% confidence interval of this indirect effect did include zero, meaning the effect was also not significant (CI 95% [-0.03;0.48]). This indicates that there was no significant mediation of behavioral resistance in the association between self-control and exercise behavior, which did not support our hypotheses.

#### Study and Work Behavior

As hypothesized, correlations between our key study and work behavior variables showed that self-control level was positively, although not significantly correlated to self-reported hours of study [*r*_(110)_ = 0.15, *p* = 0.13], and significantly negatively related to behavioral resistance toward studying [*r*_(110)_ = −0.43, *p* < 0.001]. Behavioral resistance toward studying/work and reported hours spent on studying/work were negatively related [*r*_(110)_ = −0.50, *p* < 0.001]. These associations suggest that, as hypothesized, behavioral resistance may mediate between self-control and study and work behavior and study and work behavior strength.

The indirect effect between self-control and self-reported hours of study through behavioral resistance toward studying was estimated at 0.45. The 95% confidence interval of this indirect effect did not include zero, meaning the mediation effect was significant (CI 95% [0.21; 0.73]). This indicates that self-control predicts more hours of study/work through lowered behavioral resistance. Both mediation analyses supported our hypothesis.

### Discussion

Findings demonstrate that for healthy eating and study/work behavior, behavioral resistance mediates the relationship between self-control and self-reported behavior. For these behaviors, a higher level of self-control predicted a lower level of behavioral resistance toward the specific behavior, which in turn increased the frequency or amount of desired behavior as reported by the participants. This is a second indication of our proposed underlying process explaining the positive association between higher trait self-control and self-control behavior: it suggests that people with high trait self-control experience less resistance, or aversion, to the behaviors they need to perform in order to achieve their long-term goals.

Unexpectedly, there was no significant mediation of behavioral resistance between self-control level and exercise behavior or exercise habit strength: although behavioral resistance toward exercise behavior and the actual hours of exercise as well as exercise habit strength were correlated, self-control did not predict behavior nor behavioral resistance significantly. This is surprising, since exercise is known as a typical self-control behavior and has been empirically linked to self-control in the literature multiple times (Sniehotta et al., 2005; Wills et al., 2007; Hagger et al., 2010). Therefore, it may be due to the design or assessment of exercise behavior rather than the effect being truly absent.

Study 2 is a further demonstration of how the way people perceive the aversiveness of self-control related behaviors is of importance in how their self-control level leads to performing desired behavior that is beneficial in the long run. To gain further confidence in our proposed model, Study 3 served as a conceptual replication with several methodological improvements.

## Study 3

The aim of Study 3 was to replicate the findings from Study 2 in a different sample, and to test whether a shortened version of the behavioral resistance questionnaire would have the same predictive value and reliability as the longer version used in Study 2. While Study 2 was conducted in a mainly student sample, Study 3 was conducted on the online crowdsourcing platform Mechanical Turk^1^, providing a community sample. Studying and work were not used as an area of self-control behavior in Study 3 as we anticipated a much more heterogeneous sample including participants who worked full-time, which would cloud the results. Therefore, Study 3 only included eating healthy and exercising, while assessing multiple aspects of both behaviors rather than a mere general frequency as in Study 2. For example, as the findings for exercise in Study 2 may have been clouded by participants’ different interpretations of what was meant by “exercise,” Study 3 explicitly distinguished strenuous and mild exercise while providing participants with examples of both.

Regarding the measure of behavioral resistance, in Study 3 a revised scale was used that consisted of items chosen to most accurately reflect appraisal of the task. It could be critically argued that some items used to assess behavioral resistance in Study 2 already incorporated some extent of self-regulatory failure or delay (e.g., “Even if I don’t feel like it, I still do my study work”). Therefore, the revised scale in Study 3 provides a stricter test of the role of behavioral resistance in the relation between self-control and behavior. As a final methodological improvement, the order of measurements was adapted to more adequately reflect our supposed mediation model (i.e., first self-control, then behavioral resistance, then behavioral outcomes and habits).

### Methods

#### Participants and Design

A total of 101 participants recruited from Amazon Mechanical Turk^2^ took part in this cross-sectional study. To exclude participants who filled in the survey more than once, duplicate IP addresses were excluded, leaving a sample of 81 participants (45 males, 36 females). This means that the participant sample did not meet the recommended sample size of *N* = 115 to detect a mediation effect of small/medium size with 0.8 power, unfortunately (alpha path 0.26, beta path 0.39; Fritz and MacKinnon, 2007). Mean age of the participants was 34.95 (*SD* = 11.68). The study was approved by the institution’s ethics committee.

The study had a cross-sectional design The independent variable was self-control, the dependent variables were the self-control behaviors, and the mediator was behavioral resistance toward specific behaviors. Participants received $0.50 as compensation.

#### Materials

Self-control was assessed with the Brief Self-Control Scale (Tangney et al., 2004). Details of this scale can be found in the Materials section of Study 1. The scale proved reliable with a Cronbach’s α of 0.89.

Behavioral resistance was assessed separately for healthy eating behavior and exercise behavior using three items: “[the behavior] is something I find pleasurable (reverse coded),” “if I’m honest, [the behavior] is something I’d rather not do,” and “I enjoy [the behavior] (reverse coded).” Both scales proved reliable with a Cronbach’s α of 0.94 for behavioral resistance toward exercise behavior, and a Cronbach’s α of 0.87 for behavioral resistance toward eating healthy and were answered on Likert scales ranging from 1 (strongly disagree) to 5 (strongly agree).

Eating and exercise behavior were assessed via self-reports. For healthy eating, participants were asked to report how many days of the previous week they consumed five or more portions of fruit and vegetables, how many days of the previous week they consumed deep fried foods with their meals, and on how many days in the previous week participants consumed unhealthy snacks between meals (all ranging from 0 to 7 days). For exercise behavior, participants were asked to report how many times in the previous month they performed strenuous exercise (daily, 2–3 times a week, once a week, 2–3 times a month, once a month, not during this month), how many times in the previous month they performed mild exercise (daily, 2–3 times a week, once a week, 2–3 times a month, once a month, not during this month), and how many minutes participants estimated they were physically active on a daily basis (less than 15 min a day, 15–30 min a day, 30–60 min a day, 60–120 min a day, or more than 2 h a day).

#### Procedure

Participants were invited to join an online survey. Informed consent was obtained at the start of the survey. After filling out the Brief Self-Control Scale, for each behavior, the behavioral resistance scale, and questions about the actual behavior (healthy eating and exercise) were presented to the participants. Finally, demographic information was obtained, and participants were debriefed.

### Results

**FIGURE 5:**
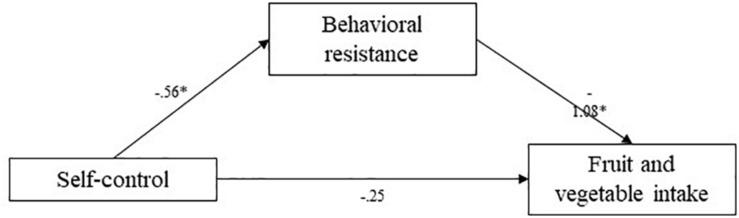
Study 3 mediation pathways including PROCESS macro (Hayes, 2012) unstandardized coefficients for associations between self-control, behavioral resistance, and fruits and vegetables. ^∗^means: significant effect.

**FIGURE 6:**
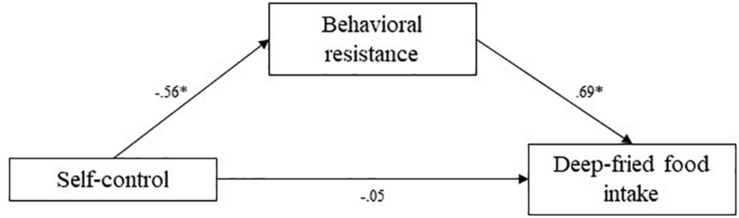
Study 3 mediation pathways including PROCESS macro (Hayes, 2012) unstandardized coefficients for associations between self-control, behavioral resistance, and deep-fried food intake. ^∗^means: significant effect.

**FIGURE 7:**
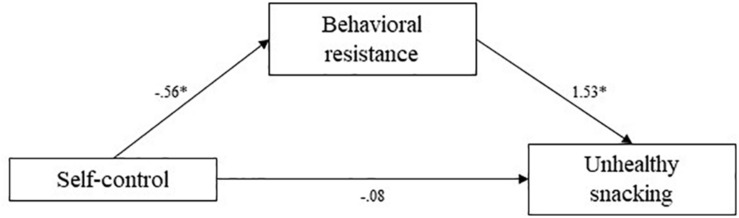
Study 3 mediation pathways including PROCESS macro (Hayes, 2012) unstandardized coefficients for associations between self-control, behavioral resistance, and unhealthy snacking. ^∗^means: significant effect.

**FIGURE 8:**
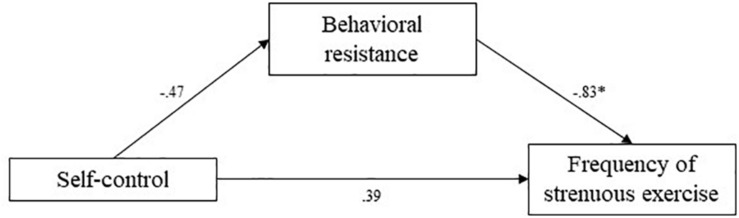
Study 3 mediation pathways including PROCESS macro (Hayes, 2012) unstandardized coefficients for associations between self-control, behavioral resistance, and frequency of strenuous exercise. ^∗^means: significant effect.

**FIGURE 9:**
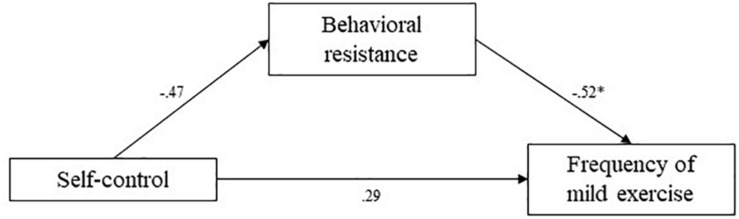
Study 3 mediation pathways including PROCESS macro (Hayes, 2012) unstandardized coefficients for associations between self-control, behavioral resistance, and frequency of mild exercise. ^∗^means: significant effect.

**FIGURE 10:**
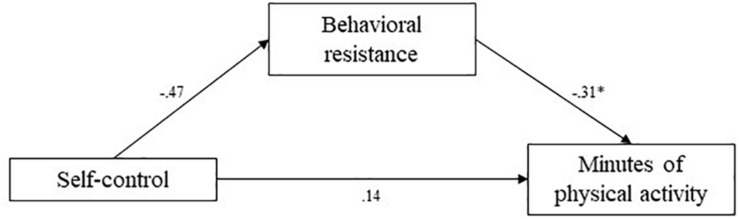
Study 3 mediation pathways including PROCESS macro (Hayes, 2012) unstandardized coefficients for associations between self-control, behavioral resistance, and minutes of physical activity. ^∗^means: significant effect.

Means and standard deviations for key variables are presented in Table 1. To test the hypotheses, correlations between the relevant variables were calculated, and mediation analyses were conducted using Hayes (2012) PROCESS macro with 1,000 bootstrap samples per analysis. Mediation pathways are depicted in Figures 5–10.

#### Healthy Eating

Table 2 displays the correlations between self-control level, behavioral resistance, and the different variables assessing self-reported healthy eating behavior.

**TABLE 2:** Correlations (*N* = 81) between self-control level, behavioral resistance, and behavioral variables of eating healthy.

	1	2	3	4	5
Self-control (1)	1	−0.40**	0.27*	−0.19^#^	−0.29**
Behavioral resistance healthy eating (2)	−0.40**	1	−0.52**	0.43**	0.69**
Fruit consumption (3)	0.27*	−0.52**	1	−0.29**	−0.39**
Deep fried food consumption (4)	−0.19^#^	0.43**	−0.29**	1	0.47**
Unhealthy snack consumption (5)	−0.29**	0.69**	−0.39**	0.47**	1

**p < 0.001, *p < 0.05, ^#^marginally significant p < 0.10.

Using the PROCESS macro (Hayes), indirect effects were estimated. The indirect effect of behavioral resistance toward eating healthy in the association between self-control and reported frequency of fruit and vegetable consumption was estimated at 0.61 (95% CI [0.26; 1.02]). The indirect effect of behavioral resistance in the association between self-control and reported frequency of deep fried food consumption was estimated at -0.38 (95% CI [-0.90; -0.07]). Finally, the indirect effect of behavioral resistance in the association between self-control and reported frequency of unhealthy snacking was estimated at -0.86 (95% CI [−1.37; −0.37]). In all cases, confidence intervals did not include zero, indicating significant mediation by behavioral resistance. Higher self-control levels predicted lower behavioral resistance toward healthy eating, which in turn predicted a higher self-reported fruit consumption, lower deep-fried food consumption, and lower unhealthy snacking consumption. These mediation analyses supported our hypotheses.

#### Exercise Behavior

Table 3 displays the correlations between self-control level, behavioral resistance, and the different variables assessing self-reported exercise behavior.

**TABLE 3:** Correlations (*N* = 81) between self-control level, behavioral resistance, and behavioral variables of exercise.

	1	2	3	4	5
Self-control (1)	1	−0.27*	0.33**	0.24*	0.18
Behavioral resistance exercise (2)	−0.27*	1	−0.65**	−0.43**	−0.36**
Strenuous exercise (3)	0.33**	−0.65**	1	0.53**	0.38**
Mild exercise (4)	0.24*	−0.43**	0.53**	1	0.50**
Minutes physically active (5)	0.18	−0.36**	0.38**	0.50**	1

**p < 0.001, *p < 0.05.

The indirect effect of behavioral resistance toward exercise in the association between self-control and reported frequency of strenuous exercise was estimated at 0.39 (95% CI [0.11; 0.75]). The indirect effect of behavioral resistance in the association between self-control and reported frequency of mild exercise was estimated at 0.25 (95% CI [0.03; 0.59]). Finally, the indirect effect of behavioral resistance in the association between self-control and reported minutes of physical activity was estimated at 0.15 (95% CI [0.03; 0.34]). In all cases, confidence intervals did not include zero, indicating significant mediation by behavioral resistance. Higher self-control levels predicted lower behavioral resistance toward exercise, which in turn predicted higher self-reported frequencies of strenuous and mild exercise, and more minutes of physical activity. These mediation analyses supported our hypotheses.

### Discussion

Study 3 again provided support for the hypothesized role of behavioral resistance as a mediator in the relation between self-control and different types of self-control behavior. It is noteworthy that, across three studies, similar results have now been found, in three different samples and for four different behavioral domains. This attests to the robustness of our conceptual model.

## General Discussion

We have reported three studies investigating behavioral resistance as a mediating factor in the association between trait self-control and goal-directed behavior. Findings from all three studies demonstrated indirect effects of behavioral resistance on the association between trait self-control and behaviors in the areas of sustainable behavior, healthy eating, exercising, and study/work.

These findings provide insight into the underlying mechanisms of successful self-control. Although behavioral resistance has been implicitly linked to self-control and self-control related behavior in literature on procrastination, the current studies provide a novel view on the relation between self-control and long-term goal-directed behaviors by directly assessing how behavioral resistance, at least partially, explains this association. Self-control is typically framed as having to do unpleasant things that can trigger feelings of resistance, like resisting nice treats, or initiating tedious tasks (Ainslie, 2013). The current studies in fact show that people with high self-control feel less resistance to these behaviors in the first place, which might mean that they are not necessarily better at inhibiting impulses or effortfully initiating unpleasant activities, but rather appraise them in a different way. People with a higher level of self-control simply do not seem to mind doing the things that need to be done in order to reach long-term goals like health and academic/career success. Hence, it becomes *easier* for them to perform behaviors that are beneficial in the long run.

These findings align with recent notions on effortless self-control (Gillebaart and De Ridder, 2015), and thereby contribute to a novel line of research scrutinizing the strategies and mechanisms associated with the successful performance of goal-directed behaviors by people with high self-control. For example, recent studies have indicated that people with higher levels of self-control experience less frequent self-control conflicts in daily life (Hofmann et al., 2012), and that people with higher levels of self-control are able to regulate emerging self-control conflicts faster, leading to smaller experienced conflicts (Gillebaart et al., 2016). The lowered behavioral resistance that we found in the current studies may be connected to these results in such a way that conflict may be more efficiently resolved, and thus experienced as smaller or easier, if resistance is lower to begin with. On the other hand, one could also construe a model in which the lower experienced behavioral resistance is a consequence of early downregulation of conflict. Either way, the current findings provide another piece of the puzzle for the notion of effortless and successful self-control.

One limitation that needs to be considered relates to the drawbacks that come with using self-reports to gauge self-control, behavioral resistance, and (self-control) behavior. Specifically, the behaviors reported on in the current studies (sustainable behavior, exercise, healthy eating, study, and work behavior) are behaviors that may be susceptible to social desirability influences. Participants may also have their own standards, norms, and expectations with regards to these behaviors, and these may influence their perception of and/or reporting on their past performance of these behaviors. Future studies may therefore include behavioral observations, but could also focus on more implicit measures of how people experience self-control behaviors. Another limitation lies in the generalizability of the findings across behavioral domains. As illustrated by the mixed findings considering exercise behavior, the results may be specific to certain, relatively concrete behaviors (e.g., “frequency of strenuous exercise,” with a clear definition of strenuous exercise), and may be less clear-cut when the behavior is framed more abstractly (e.g., “hour of exercise,” which is subject to interpretation of what exercise entails).

Importantly, due to the cross-sectional nature of the designs, the current studies do not allow conclusions regarding the causality of the associations between self-control, resistance and behavior. We cannot rule out that the supposed causality (i.e., lower behavioral resistance *leading to* better behavioral performance) is in fact reverse (e.g., more frequent desired behavior leading to lower resistance), although it is relevant to note that in the procrastination literature task aversiveness is clearly considered a predictor, rather than a result, of task performance. Future studies should more clearly investigate the causal chain, for example by prospectively assessing a novel behavior after an initial assessment of behavioral resistance, as a function of self-control. Furthermore, it would be interesting to investigate *why* people with higher trait self-control feel lower resistance toward goal-directed behaviors. One suggestion could be to combine measures of behavioral resistance with measures of experienced conflict, and conflict resolution (Gillebaart et al., 2016) to get more insight into a chain of events that captures the whole behavioral process. Another suggestion could be to focus on motivational orientation: A recent study showed that people with high self-control are more “promotion focused” compared to people with lower self-control (Cheung et al., 2014), yielding an advantageous perspective on goal-directed behaviors in the sense that they focus more on future gains rather than current “losses” (e.g., tediousness of a task), which may affect the amount of resistance they experience toward these behaviors or tasks. Finally, considering the novelty of the behavioral resistance concept, future studies should aim to map how it is distinct from, or relates to, other concepts like intrinsic and extrinsic motivation.

A third opportunity for future research lies in the application of the current findings in order to support people with lower levels of self-control. Low self-control is associated with an array of negative outcomes, including obesity and substance abuse (Tangney et al., 2004; De Ridder et al., 2012). From the reported findings, it is apparent that the way in which aversive behaviors are appraised is a relevant factor, co-determining whether that behavior is eventually carried out. People with lower levels of self-control experience high behavioral resistance, which they would need to overcome in order to perform the desired behaviors. Based on the current studies, we speculate that it may be possible to change people’s appraisal of the behavior, lowering behavioral resistance. This may lead to improved performance, enabling people with lower self-control to pursue their long-term goals. Based on our findings, this may be true for both initiatory and inhibitory self-control behaviors (De Ridder et al., 2011). Self-control can be necessary to initiate certain behaviors (e.g., initiating exercise behavior), but is usually seen as having a strong inhibitory component (e.g., inhibiting unhealthy eating behavior). Although intuitively, one may associate behavioral resistance with initiation of behavior rather than inhibition, our findings on lower frequencies (i.e., inhibition) of deep-fried food consumption and unhealthy snacking show similar patterns of self-control and behavioral resistance as the initiation-based behaviors.

## Conclusion

In conclusion, results from three studies demonstrate that level of self-control is associated with long-term goal-directed behaviors in the areas of sustainable behavior, healthy eating, exercise, and study/work. This association is, at least partially, explained by how much resistance people feel toward these behaviors: People with higher levels of self-control experience lower levels of resistance, which in turn is associated with frequency or amount of goal-directed behavior. These findings are in line with a perspective on self-control encompassing not only effortful inhibition of impulses, but rather focusing on the effortless strategies that can facilitate self-control success.

## Data Availability Statement

Data and analysis scripts will be made public upon acceptance for publication and will be accessible via https://osf.io/cd9j6/.

## Ethics Statement

The studies involving human participants were reviewed and approved by Faculty of Social and Behavioral Sciences Ethics Review Board. The participants provided their written informed consent to participate in this study.

## Author Contributions

MG and FK contributed significantly to theory formation, research questions, study design, data analysis, and reporting of the research.

## Conflict of Interest

The authors declare that the research was conducted in the absence of any commercial or financial relationships that could be construed as a potential conflict of interest.

## Footnotes

1 www.mturk.com

## Appendix

Study 2 behavioral resistance scale (translated from Dutch)

Sometimes I don’t have the energy to [X]

Just the thought of doing [X] makes me want to not do it

I tend to delay doing [X]

Sometimes I just do something easier than [X] because I don’t feel like doing [X]

Sometimes I allow myself to not do [X]

I experience doing [X] as unpleasant

Sometimes I delay doing [X] because I don’t feel like doing it.

I like doing [X] (reverse coded)

I look forward to doing [X] (reverse coded)

I do [X] even if I’m low on energy (reverse coded).
